# The mean field market model revisited

**DOI:** 10.1007/s13385-025-00408-9

**Published:** 2025-02-07

**Authors:** Manuel Hasenbichler, Wolfgang Müller, Stefan Thonhauser

**Affiliations:** Institute of Statistics, Graz University of Technology, Kopernikusgasse 24/III, 8010 Graz, Styria Austria

**Keywords:** Overnight rates, LIBOR market model, Solvency II, McKean–Vlasov process, 60G51, 60H10, 60J70, 91G05, 91G30

## Abstract

In this paper, we present an alternative perspective on the mean-field LIBOR market model introduced by Desmettre et al. (Int J Theor Appl Finance, 2022. https://doi.org/10.1142/S0219024922500054). Our novel approach embeds the mean-field model in a classical setup, but retains the crucial feature of controlling the term rate’s variances over large time horizons. This maintains the market model’s practicability, since calibrations and simulations can be carried out efficiently without nested simulations. In addition, we show that our framework can be directly applied to model in-arrear term rates derived from SOFR, ESTR or other nearly risk-free overnight short-term rates—a crucial feature since many IBOR rates are gradually being replaced. These results are complemented by a calibration study and some theoretical arguments which allow to estimate the probability of unrealistically high rates in the presented market models.

## Introduction and motivation

In 2015, the Commission of the European Union amended the Solvency II^1^ Directive issued in 2009, which regulates all insurance businesses in the European Union (cf. [3]). As a result, insurance companies operating in the European Union must assign market-consistent values to all balance sheet positions since the new regulation came into force in 2016. For the evaluation of liabilities—particularly in the context of life insurance contracts—this requires the market-consistent simulation of interest rates to determine the present value of future benefits, the so called *best estimates*, which are agreed on in the corresponding insurance contracts (cf. [5, Sec. 1]). For the presentation and study of general cashflow models underlying insurance best estimates the interested reader is referred to [5, 6].

Meanwhile, the Forward market model (or LIBOR market model), which models term rates for a finite and predetermined number of tenors, has gained increasing popularity over the last decades as it can be easily calibrated to market data. However, life insurance contracts can have maturities of up to 60 years, while liquid caplet and swaption prices, both of which are commonly used to calibrate the models, are typically only traded for maturities of 20 to 30 years in the future. For this reason, practitioners have to extrapolate from the model over a long period relative to the time horizon for which reliable market data is available. This practice, however, sometimes leads to the so-called phenomenon of *term rate blow-ups*, especially if the model is calibrated in more turbulent market environments. In this piece of work, term rate blow-ups refer to the event where, at a certain future point in time, a significant proportion (e.g. more than 5%) of simulated term rates are unrealistically high (e.g. higher than 70%). Such issues, arising from both the general regulatory framework and from the application of this type of model in this context, have already been examined (e.g. cf. [2, 12]). In [2], Desmettre et al. propose to extend the classical Forward market model’s dynamics to mean-field stochastic differential equations (MF-SDEs). This allows them to damp the coefficients driving the model’s dynamics if the term rates’ variances and hence the probability of blow-ups become too high.

To simulate forwards rates form their model, Desmettre et al. apply the particle approximation approach. However, this standard technique for the simulation of stochastic processes driven by MF-SDEs involves computationally expensive and time-consuming nested simulations and hence can be hardly applied for calibration purposes in practice. In Sect. 2 we consider models where the coefficients of the MF-SDEs depend only on time and a finite number of the rates’ moments as opposed to their entire distribution. This allows us to calculate the moments of the term rates as solutions of ODEs. Given these moments, the model is then a classical Forward market model, which allows practitioners to simulate from and calibrate the model in a time-efficient manner. Furthermore, we describe a class of models for which these ODEs can be solved analytically, resulting in a very tractable class of models.

In addition, we apply the ideas introduced in [7, 8] and formulate the results for in-arrears or “backward-looking term rates” that are e.g. stripped from overnight short-term rates such as SONIA, SOFR or ESTR too. In order to avoid arbitrariness (also cf. [12, Sec. 3.2.4]), special emphasis is placed on the development of criteria to avoid overzealous reduction of the term rate’s variances that is inconsistent with market observations. In the appendix, we present a technique which allows us to a-priori estimate the effectiveness of a chosen dampening approach.

We start by introducing the stochastic basis that is adapted from [2]. Let ${0 = T_0< T_1< \cdots< T_N < \infty }$, $N \in \mathbb {N}$ be a prespecified set of settlement dates, $\left( \Omega ,\mathcal {A},\mathcal {F},\mathbb {Q}^N\right)$ be a filtered probability space supporting Brownian motion and suppose that the filtration $\mathcal {F}$ fulfills the usual conditions^2^ with $\mathcal {A} = \mathcal {F}_{T_N}$. Moreover, denote $\mathbb {Q}^i$ the $T_i$-forward measure for all $1\le i\le N$, $\mathcal {P}\left( \mathbb {R}\right)$ the set of probability measures on $\mathbb {R}$ and define

$$\begin{aligned} \mathcal {P}_k\left( \mathbb {R}\right) = \left\{ \mu \in \mathcal {P}\left( \mathbb {R}\right) : \int _\mathbb {R} x^k \, \textrm{d}\mu (x) < \infty \right\} , \quad k \in \mathbb {N}. \end{aligned}$$

As proposed in [2], we model the term rates ${\{F_i\}_{i=1}^N:= \{t \mapsto F(t,T_{i-1},T_i)\}_{i=1}^N}$ associated with this tenor structure by

1 $$\begin{aligned} {\left\{ \begin{array}{ll} \textrm{d}F_i(t) = F_i(t)\sigma _i\left( t, \mu _t^i\right) ^T \textrm{d}W_t^i \quad 0 \le t \le T_{i-1}, \\ F_i(0) = F\left( 0,T_{i-1},T_i\right) > 0, \end{array}\right. } \end{aligned}$$

if the term rates are forward-looking (e.g. IBORs such as EURIBOR or LIBOR) and

2 $$\begin{aligned} {\left\{ \begin{array}{ll} \textrm{d}F^B_i(t) = F^B_i(t)\sigma ^B_i\left( t, \mu _t^i\right) ^T \textrm{d}W_t^i \quad 0 \le t \le T_i, \\ F^B_i(0) = F^B\left( 0,T_{i-1},T_i\right) > 0, \end{array}\right. } \end{aligned}$$

if the term rates are backward-looking (e.g. term rates stripped from overnight short-term rates such as SONIA, SOFR or ESTR). Hereby, $W^i$ is $\mathbb {Q}^i$ *d*-dimensional Brownian motion, $\mu _t^i \in \mathcal {P}_k\left( \mathbb {R}\right)$ is the law of $F_i(t)$ respectively $F^B_i(t)$ under $\mathbb {Q}^i$ and

$$\begin{aligned} \sigma _i: [0,T_{i-1}]\times \mathcal {P}_k\left( \mathbb {R}\right) \rightarrow \mathbb {R}^d \end{aligned}$$

in the first case and

$$\begin{aligned} \sigma ^B_i: [0,T_i]\times \mathcal {P}_k\left( \mathbb {R}\right) \rightarrow \mathbb {R}^d \end{aligned}$$

in the latter case. In this paper, we mostly consider $d = N$ for the purpose of simplicity. If we denote by $R(T_{i-1},T_i) = F_i(T_{i-1})$ the forward-looking interest that is accrued in $[T_{i-1},T_i]$ and by $R^B(T_{i-1},T_i) = F^B_i(T_i)$ the backward-looking interest that is accrued in $[T_{i-1},T_i]$, backward and forward-looking interest rates conceptionally only differ in their measurability in lognormal market models. In fact, Lyashenko and Mercurio show the following proposition in [8]:

### Proposition 1.1

[8, Sec. 2.4] Let $0< S < T$. Then, $F^B(t,S,T) = F(t,S,T) \quad \forall \, t \in [0,S]$.

Thus, we refrain from stating analogous results and omit the superscript *B* in the following.

As in the classical theory on the Forward market model, the various forward measures are linked by the change of measure processes

$$\begin{aligned} \frac{\textrm{d}\mathbb {Q}^{i-1}}{\textrm{d}\mathbb {Q}^{i}}  &   = \exp \Bigg (\int _0^\cdot \frac{\Delta _i F_i(s)}{1 + \Delta _i F_i(s)} \left( \sigma _i\left( s,\mu _s^i\right) \right) ^T\textrm{d}W_s^i \\    &   - \frac{1}{2}\int _0^\cdot \left| \frac{\Delta _i F_i(s)}{1 + \Delta _i F_i(s)} \sigma _i\left( s,\mu _s^i\right) \right| ^2\textrm{d}s \Bigg ), \end{aligned}$$

assuming that the term rates $\{F_i\}_{i=1}^N$ exist on $\left( \Omega ,\mathcal {A},\mathcal {F}\right)$. We recall that this ensures that there exists a probability measure $\mathbb {Q}^* \sim \mathbb {Q}^N$ such that the price at time $T_k$ of any $\mathcal {F}_{T_N}$-measurable and attainable claim *X* is

$$\begin{aligned} \pi _X(T_k) = B^*(T_k) \, \mathbb {E}_{\mathbb {Q}^*}\left[ \frac{X}{B^*(T_N)}\bigg |\mathcal {F}_{T_k}\right] \quad \forall \, 1 \le k \le N \end{aligned}$$

where

$$\begin{aligned} {\left\{ \begin{array}{ll} B^*(0) = 1, \\ B^*(T_i) = \left( 1 + \Delta _i F_i(T_i)\right) )B^*(T_{i-1}), \quad \forall \, 1 \le i \le N \end{array}\right. } \end{aligned}$$

is the discrete-time implied bank account process (e.g. c.f. [4, Chpt. 11]). In the case of backward-looking term rates, this bank account process is no-longer previsible but still monotonically increasing and adapted with respect to $\left\{ \mathcal {F}_{T_i}\right\} _{i=1}^N$.

## Theoretical considerations

If the instantaneous volatilities $\sigma _i\left( t,\mu _t^i\right)$ are only functions of time and a finite number of moments of the *i*th term rate and do not depend on the entire distribution $\mu ^i$ ${\forall \, 1 \le i \le N}$, damping effects can already be achieved in the classical Forward market model framework, as Theorem 2.1 below suggests. In particular, it proposes a method that allows practitioners to achieve the same damping effects as presented by [2] without performing time-consuming nested simulations. Nonetheless, the ordinary differential equations (ODEs) arising from this method need to be solved numerically. Therefore we additionally describe a class of models for which these ODEs have explicit solutions. This allows the easy-to-implement simulation of forward- and backward-looking term rates.

### Existence and uniqueness

We show that the existence of the term rates $\left\{ F_i\right\} _{i=1}^N$ in our special model is intertwined with the existence of solutions to a set of ODEs.

#### Theorem 2.1

Suppose that for all $1 \le i \le N$ the function $\ \displaystyle {\sigma _i: [0,T_i] \times \mathcal {P}_k\left( \mathbb {R}\right) \rightarrow \mathbb {R}^d}$ has the form

$$\begin{aligned} \sigma _i\left( t,\mu _t^i\right) = \lambda _i\left( t,\mathbb {E}_{\mathbb {Q}^i}\left[ \left( F_i(t)\right) ^2\right] ,\dots ,\mathbb {E}_{\mathbb {Q}^i}\left[ \left( F_i(t)\right) ^k\right] \right) \end{aligned}$$

where ${\lambda _i: [0,T_i] \times \left( \mathbb {R}^+\right) ^{k-1}} \rightarrow \mathbb {R}^d$ is measurable and its length $\left| \lambda _i\right|$ is piecewise continuous in every coordinate and non-zero. Then, the following statements hold:

1. Let $\left( F_1,\dots ,F_N\right)$ be a solution of (2) on $\left( \Omega ,\mathcal {F},\mathbb {Q}^N\right)$ and suppose $t \mapsto \left| \sigma _i(t,\mu _t^i)\right|$ is bounded on $[0,T_i]$. Then, for every $1 \le j \le N$ the function $\displaystyle \psi _i^j(t):= \mathbb {E}_{\mathbb {Q}^i}\left[ F_i(t)^j\right]$ is the unique continuous and piecewise differentiable solution of
  3 $$\begin{aligned} {\left\{ \begin{array}{ll} \frac{\textrm{d}}{\textrm{d}t}\psi _i^j(t) = \frac{j(j-1)}{2} \psi _i^j(t) \left| \lambda _i(t,\psi _i^2(t),\dots ,\psi _i^k(t))\right| ^2 \quad \left( t \in \mathcal {D}_i\cap (0,T_i)\right) , \\ \psi _i^j(0) = \left( F_i(0)\right) ^j, \end{array}\right. } \end{aligned}$$
  where $\displaystyle \mathcal {D}_i:= \left\{ t : \, \psi _i^j \text { is differentiable in } t \text { for all } 2 \le j \le k\right\}$ for all $1 \le i \le N$.
2. Suppose there exist continuous and piecewise differentiable $\left( \psi _i^j\right) _{j=2}^k$ for all ${1 \le i \le N}$ such that for all *i*, *j* the function ${\psi _i^j: [0,T_i] \rightarrow \mathbb {R}^+}$ satisfies
  4 $$\begin{aligned} {\left\{ \begin{array}{ll} \frac{\textrm{d}}{\textrm{d}t}\psi _i^j(t) = \frac{j(j-1)}{2} \psi _i^j(t) \left| \lambda _i(t,\psi _i^2(t),\dots ,\psi _i^k(t))\right| ^2 \quad \left( t \in \mathcal {D}_i\cap (0,T_i)\right) , \\ \psi _i^j(0) = \left( F_i(0)\right) ^j, \end{array}\right. } \end{aligned}$$
  where $\displaystyle \mathcal {D}_i:= \left\{ t : \, \psi _i^j \text { is differentiable in } t \text { for all } 2 \le j \le k\right\}$. Then, for all $1 \le i \le N$ there exists an unique strong solution to (2) on $\left( \Omega ,\mathcal {F},\mathbb {Q}^N\right)$ and for all *i*, *j*
  5 $$\begin{aligned} \mathbb {E}_{\mathbb {Q}^i}\left[ F_i(t)^j\right] = \psi _i^j(t) \quad 0 \le t \le T_i.\end{aligned}$$

#### Proof

1. By construction, for $t \le T_i$
  $$\begin{aligned} F_i(t)^j = F_i(0)^j \, \exp \left( j \, \int _0^t\sigma _i(s,\mu _s^i) \textrm{d}W_s^i - \frac{j}{2} \int _0^t\left| \sigma _i(s,\mu _s^i)\right| ^2 \textrm{d}s\right) \end{aligned}$$
  where
  $$\begin{aligned} \sigma _i\left( t,\mu _t^i\right) = \lambda _i\left( t,\mathbb {E}_{\mathbb {Q}^i}\left[ F_i(t)^2\right] ,\dots ,\mathbb {E}_{\mathbb {Q}^i}\left[ F_i(t)^k\right] \right) . \end{aligned}$$
  For $2 \le j \le k$ set $\psi _i^j(t):= \mathbb {E}_{\mathbb {Q}^i}\left[ F_i(t)^j\right]$. Then,
  6 $$\begin{aligned} \psi _i^j(t) = F_i(0)^j \exp \left( \frac{j(j-1)}{2}\int _0^t \left| \lambda _i\left( s,\psi _i^j(s),\dots ,\psi _i^k(s)\right) \right| ^2 \textrm{d}s\right) . \end{aligned}$$
  Since $t \mapsto \sigma _i(t,\mu _t^i)$ is bounded on $[0,T_{i}]$, $\psi _i^j$ are continuous on $\left[ 0,T_{i}\right]$. In addition, $\left( \psi _i^j\right) _{j=2}^k$ are piecewise differentiable since $\left| \lambda _i\right|$ is piecewise continuous in every coordinate. Differentiating the above expression w.r.t. $t \in \mathcal {D}_i$ yields (3).
2. Define $\displaystyle \tilde{\sigma }_i(t):= \lambda _i\left( t,\psi _i^2(t),\dots ,\psi _i^k(t)\right)$ for all $1 \le i \le N$ where $\left( \psi _i^j\right) _{j=2}^k$ are the continuous and piecewise differentiable functions that solve (4). It is well-known (e.g. c.f. [9], Ch. 12.4) that there exist unique strong solutions of
  $$\begin{aligned} {\left\{ \begin{array}{ll} \textrm{d}F_i(t) = F_i(t)\tilde{\sigma }_i(t)^T \textrm{d}W_t^i \quad 0 \le t \le T_i, \\ F_i(0) = F_i\left( 0,T_{i-1},T_i\right) > 0, \end{array}\right. } \end{aligned}$$
  on $\left( \Omega ,\mathcal {F},\mathbb {Q}^N\right)$ since the $\tilde{\sigma }_i$ are bounded and piecewise continuous on $[0,T_{i}]$. It remains to show that $\psi _i^j(t) = \mathbb {E}_{\mathbb {Q}^i}\left[ F_i(t)^j\right]$ on $[0,T_{i}]$ for all *i* and *j*.
  By construction,
  $$\begin{aligned} F_i(t)^j = F_i(0)^j \, \exp \left( j \, \int _0^t \tilde{\sigma }_i(s) \textrm{d}W_s^i - \frac{j}{2} \int _0^t\tilde{\sigma }_i(s)^2 \textrm{d}s\right) \end{aligned}$$
  and therefore
  7 $$\begin{aligned} \mathbb {E}_{\mathbb {Q}^i}\left[ F_i(t)^j\right] = F_i(0)^j \exp \left( \frac{j(j-1)}{2}\int _0^t \left| \tilde{\sigma }_i(s)\right| ^2 \textrm{d}s\right) . \end{aligned}$$
  Since the right hand side of this equation is continuous and piecewise differentiable in *t*, so is $\mathbb {E}_{\mathbb {Q}^i}\left[ F_i(t)^j\right]$. What is more,
  $$\begin{aligned} \mathbb {E}_{\mathbb {Q}^i}\left[ F_i(0)^j\right] = F_i(0)^j = \psi _i^j(0) \end{aligned}$$
  and note that $\mathbb {E}_{\mathbb {Q}^i}\left[ F_i(t)^j\right]$ is differentiable in *t* iff $\left| \lambda _i\left( t,\psi _i^2(t),\dots ,\psi _i^k(t)\right) \right| ^2$ is continuous in *t*. Hence, $\displaystyle \mathcal {D}_i = \left\{ t : \, \mathbb {E}_{\mathbb {Q}^i}\left[ F_i(t)^j\right] \text { is differentiable in } t\right\} .$ By differentiating (7) in $\in \mathcal {D}_i$, one finds that $\mathbb {E}_{\mathbb {Q}^i}\left[ F_i(0)^j\right]$ is the unique solution of (4).

$$\square $$

#### Remark 2.1

Equation (6) shows that the damping of higher moments ($k > 2$) produces the same damping effect as the damping of the second moments of the forward rates.

We primarily strive to present a method to mitigate interest rate blow-ups when employing the Forward market model. By the last remark, it is sufficient to consider the case $k=2$ of Theorem 2.1. Similar to the approach developed in [2], we use the term rates’ total variance $\phi _i$ instead of $\psi _i^2$ as a control variable to reduce blow-ups. $\phi _i$ classically arises in option trading and corresponds to $T_i \cdot \left( \sigma _i^{BS}\right) ^2$, where $\sigma _i^{BS}$ is the Black implied $F_i$-caplet volatility. In our log-normal model setting,

8 $$\begin{aligned} \phi _i(t) = \log \left( \frac{\psi _i^2(t)}{F_i(0)^2}\right) = \log \left( \frac{\mathbb {E}_{\mathbb {Q}^i}\left[ F_i(t)^2\right] }{F_i(0)^2}\right) = \int _0^t \left| \sigma _i(s,\mu _s^i)\right| \textrm{d}s. \end{aligned}$$

This is just a reparametrisation. Note that in terms of $\phi _i$, Eq. (4) is equivalent to

9 $$\begin{aligned} {\left\{ \begin{array}{ll} \frac{\textrm{d}}{\textrm{d}t}\phi _i(t) = \frac{1}{\psi _i^2(t)}\frac{\textrm{d}}{\textrm{d}t}\psi _i^2(t) = |\sigma _i(t,\mu _t^i)|^2 \quad \left( t \in \mathcal {D}_i\cap (0,T_i)\right) , \\ \phi _i(0) = 0. \end{array}\right. } \end{aligned}$$

We use this reparametrisation to describe a class of models where (9) and hence (4) have explicit solutions.

#### Corollary 2.1.1

Suppose that for all ${1 \le i \le N}$ the function $\displaystyle \sigma _i: [0,T_i] \times \mathcal {P}_2\left( \mathbb {R}\right) \rightarrow \left( \mathbb {R}^+\right) ^d$ has the form

$$\begin{aligned} \sigma _i\left( t,\mu _t^i\right) = g_i(t) f_i\left( \phi _i(t)\right) v_i\left( t,\phi _i(t)\right) \end{aligned}$$

with $\phi _i$ as in (8), where $g_i: [0,T_i] \rightarrow \mathbb {R}^+$, $f_i: \mathbb {R}_0^+ \rightarrow \mathbb {R}^+$ are left-continuous as well as piecewise continuous and $v_i: [0, T_i] \times \mathbb {R}_0^+ \rightarrow \mathcal {S}^{d-1} = \left\{ x \in \mathbb {R}^d : \, |x |_2 = 1 \right\}$ is left-continuous. In addition, assume that

10 $$\begin{aligned} \int _0^{T_{i}} g_i(s)^2 \textrm{d}s \le \int _0^\infty \frac{1}{f_i(z)^2} \textrm{d}z \quad \forall \, 1 \le i \le N. \end{aligned}$$

Then, $F_i$ exists as an unique strong solution to (2). Furthermore,

11 $$\begin{aligned} \phi _i(t) = V_i\left( \int _0^t g_i(s)^2 \textrm{d}s\right) \end{aligned}$$

where

$$\begin{aligned} V_i^{(-1)}(x) = \int _0^x f_i(z)^{-2} \textrm{d}z. \end{aligned}$$

#### Proof

By assumption, $f_i>0$ and so, $x \mapsto \int _0^x f_i(z)^{-2} \textrm{d}z$ is well-defined, continuous and strictly monotonically increasing. Thus, its inverse ${V_i: [0,y_i) \rightarrow [0,\infty )}$ with ${y_i:= \int _0^\infty f_i(z)^{-2} \textrm{d}z \le \infty }$ exists and, given (10), is well-defined on $\left[ 0,\int _0^{T_i} g_i(s)^2 \textrm{d}s\right]$. Separation of variables shows that (11) solves (9). Then, Theorem (2.1) yields the claim. $\square$

#### Remark 2.2

While the $\left\{ g_i\right\} _{i=1}^N$ govern the principle volatility structure over time and are as such calibrated to market data as will be shown in Sect. 3.1, the $\left\{ f_i\right\} _{i=1}^N$ are responsible for damping whenever the control variables $\left\{ \phi _i\right\} _{i=1}^N$ become too large. The $\left\{ v_i\right\} _{i=1}^N$, on the other hand, determine the term rates’ correlations. Thus, the $\left\{ g_i\right\} _{i=1}^N$ are referred to as *principle factors*, the $\left\{ v_i\right\} _{i=1}^N$ are called *correlation factors* and the $\left\{ f_i\right\} _{i=1}^N$ are referred to as *damping factors* of the instantaneous volatilities $\left\{ \sigma _i(t,\mu _t^i)\right\} _{i=1}^N$ respectively. Note that $f_i \equiv 1$ ${\forall \, 1 \le i \le N}$ yields the classical Forward market model framework.

### Total implied variance structures

We need to choose $\left\{ f_i\right\} _{i=1}^N$ in Corollary 2.1.1 in a way such that we can control the damping of the total implied variances $\left\{ \phi _i\right\} _{i=1}^N$. We achieve this by specifying a suitable class of functions for $\left\{ V_i\right\} _{i=1}^N$ in (11) which allows the easy and explicit computation of $\mathbb {E}_{\mathbb {Q}^i}\left[ F_i(t)^2\right]$. To this end, we start with three definitions.

#### Definition 2.1

$k: \mathbb {R}_0^+ \rightarrow \mathbb {R}_0^+$ is a *(total implied variance structure) segment* if

1. *k* is strictly monotonically increasing and concave or convex,
2. *k* is continuously differentiable on $\mathbb {R}^+$,
3. $$k(0) = 0$$.

#### Definition 2.2

A bijective map $V: \mathbb {R}^+_0 \rightarrow \mathbb {R}^+_0$ is *induced by segments* $\left( k_i\right) _{i=0}^M$ for some $M \in \mathbb {N}_0$ if there exist strictly monotonically increasing *(total implied variance) thresholds* ${0 = \tau _0< \cdots < \tau _{M+1}=\infty }$ such that

$$\begin{aligned} V(y) = \sum _{i=0}^{M} \mathbb {1}_{[\tau _i,\infty )}(y) \, k_i(y \wedge \tau _{i+1} - \tau _i). \end{aligned}$$

**Fig. 1:**
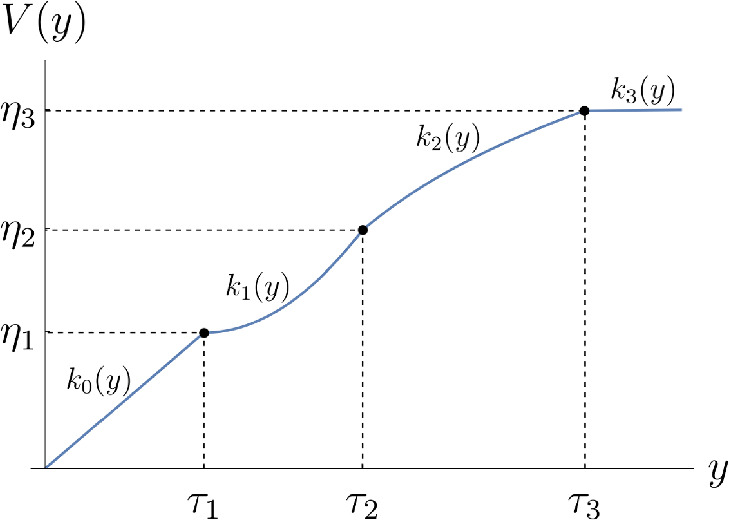
Total implied volatility structure, exemplary plot

We call such a function *V* *total implied variance structure* (e.g. c.f. Fig. 1). Note that *V* is continuous and piecewise differentiable on ${\mathcal {D}_V:= \mathbb {R}_0^+\backslash \{\tau _i\}_{i=0}^{M}}$.

#### Definition 2.3

**Fig. 2:**
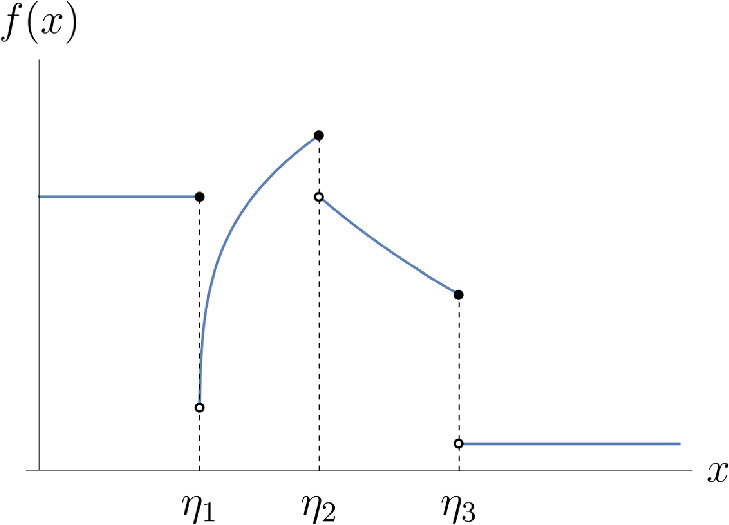
Damping factor, exemplary plot

A left-continuous function $f: \mathbb {R}_0^+ \rightarrow \mathbb {R}^+$ is called a *(damping) factor* if there exist $0 = \eta _0< \dots< \eta _M < \infty$ for some ${M \in \mathbb {N}_0}$ if *f* is continuous and either monotonically increasing or monotonically decreasing on each $(\eta _i,\eta _{i+1}]$ for ${0 \le i \le M-1}$ and on $(\eta _M,\infty )$ (e.g. c.f. Fig. 2).

As a direct consequence of Definitions 2.2 and 2.3, there exists a damping factor *f* for every total implied variance structure *V* such that $V^{(-1)} = \int _0^\cdot f(z)^{-2} \textrm{d}z$ and vice-versa. Indeed, given a total implied variance structure *V* as in Definition 2.2, one such damping factor *f* is

12 $$\begin{aligned} f(x)&= \mathbb {1}_{\{0\}}(x) f_0 + \sum _{i=0}^{M-1} \mathbb {1}_{(\eta _i,\eta _{i+1}]}(x)\left( \left[ \frac{\textrm{d}}{\textrm{d}x}k_i^{(-1)}\right] \left( x-\eta _i\right) \right) ^{-\frac{1}{2}} \nonumber \\&\quad + \mathbb {1}_{(\eta _M,\infty )}(x)\left( \left[ \frac{\textrm{d}}{\textrm{d}x}k_M^{(-1)}\right] \left( x-\eta _M\right) \right) ^{-\frac{1}{2}} \end{aligned}$$

with $\eta _i:= V(\tau _i)$ $\forall 1 \le i \le M$ and some $f_0 \in \mathbb {R}^+$. Usually, $f_0:= f(0+)$ and we will refer to this particular choice as *the associated* damping factor.

In this paper, the focus is on total implied variance structures that damp the term rates’ instantaneous volatilities and consist of at most three different concave segments. Furthermore, we choose the same total implied volatility structure for each term rate to extrapolate the given market data. In particular, we always set^3^$k_0 = \text {id}_{\mathbb {R}_0^+}$ and $\tau _1 > 0$ for reasons that we explain in more detail in Sect. 2.2.1.

However, we note that parameterised segments such as $\left\{ y \mapsto a_j y^{p_j}\right\} _{j = 1}^L$ with $a_j, p_j > 0$ for every $1 \le j \le L$ can be applied to fit any strictly increasing total implied volatility structure without the need to individually adjust the term rates’ instantaneous volatilities by multiplicative constants. This is especially important when extrapolating market data. In this way, our framework also extends the literature on market model calibration.

Two examples of total implied variance structures and their associated damping factors are given below. Of course, these basic structures can be combined to more complex ones.

#### Remark 2.3

Corollary 2.1.1 shows that we have to consider both, *V* and $V^{(-1)}$, in the computation of $\left\{ \phi _i\right\} _{i=1}^N$. As a result, easily invertible segments $k_0, \dots , k_M$ will be chosen in practice.

#### Example 2.1

(Adapted from [2] by Desmettre et al.)

Let $\tau _1 = \tau > 0$ and $k_1(y) = k(y) = \frac{\tau }{2} \log \left( 1 + \frac{2 y}{\tau }\right)$. This is a natural choice as *k* is strictly monotonically increasing, concave, continuously differentiable and satisfies $k(0) = 0$ as well as ${k'(0) = 1}$. Thus, *k* is a segment that, together with the threshold $\tau$, induces the continuously differentiable total implied variance structure $V: \mathbb {R}_0^+ \rightarrow \mathbb {R}_0^+$ given by

$$\begin{aligned} V(y) = {\left\{ \begin{array}{ll} y &  \text {if } y \le \tau \\ \tau + k(y-\tau ) &  \text {if } \tau \le y \end{array}\right. } = {\left\{ \begin{array}{ll} y &  \text {if } y \le \tau , \\ \tau + \frac{\tau }{2} \, \log \left( \frac{2 y}{\tau } - 1\right) &  \text {if } y > \tau . \end{array}\right. } \end{aligned}$$

By (12), its associated damping factor is

$$\begin{aligned} f(x) = \mathbb {1}_{[0,\tau ]}(x) + \mathbb {1}_{(\tau ,\infty )}(x)\exp \left( -\frac{x-\tau }{\tau }\right) = \exp \left( -\frac{\left( x-\tau \right) ^+}{\tau }\right) . \end{aligned}$$

#### Example 2.2

(Pseudo-volatility freeze) Let $\tau > 0$, $\epsilon , \delta \in [0,1)$ and

$$\begin{aligned} f(x) = {\left\{ \begin{array}{ll} 1 &  \text {if } x \le \tau (1-\delta ), \\ 1 - (x - (1 - \delta )\tau )\frac{1 - \epsilon }{\delta \tau } &  \text {if } \tau (1-\delta )< x \le \tau , \\ \epsilon &  \text {if } \tau < x. \end{array}\right. } \end{aligned}$$

Again, *f* is a damping factor and

$$\begin{aligned} H(x) = \int _0^x f(z)^{-2} \textrm{d}z = {\left\{ \begin{array}{ll} x &  \text {if } x \le \tau (1-\delta ), \\ (1 - \delta )\tau + \frac{\delta \tau (x - (1 - \delta ) \ \tau )}{\tau - x (1 - \epsilon ) - (1 - \delta ) \epsilon \tau } &  \text {if } \tau (1-\delta )< x \le \tau , \\ \frac{x - (1 - \epsilon (\delta + \epsilon - \delta \epsilon )) \tau }{\epsilon ^2} &  \text {if } \tau < x. \end{array}\right. } \end{aligned}$$

Its inverse is

$$\begin{aligned} V(y) = {\left\{ \begin{array}{ll} y &  \text {if } y \le \tau (1-\delta ), \\ -\frac{\tau (y - y (1 - \delta ) \epsilon - (1 - \delta )^2 (1 - \epsilon ) \tau )}{\tau - y (1 - \epsilon ) - \delta (2 - \epsilon ) \tau - \epsilon \tau } &  \text {if } \tau (1-\delta )< y \le \tau + \delta \left( \frac{1}{\epsilon } - 1\right) \tau , \\ \tau + \epsilon (y \epsilon - \delta (1 - \epsilon ) \tau - \epsilon \tau ) &  \text {if } \tau + \delta \left( \frac{1}{\epsilon } - 1\right) \tau < y. \end{array}\right. } \end{aligned}$$

Here, $\tau _1 = (1-\delta )\tau$, $\tau _2 = \tau + \delta \left( \frac{1}{\epsilon } - 1\right) \tau$ are the total implied variance structure thresholds and

$$\begin{aligned} k_1(y) = \frac{y \delta \tau }{y(1-\epsilon ) + \delta \tau }, \quad k_2(y) = y \epsilon ^2 \end{aligned}$$

are the corresponding segments. This damping method is referred to as pseudo-volatility freeze since it mimics a volatility freeze for $\epsilon<< 1$ and $\delta<< 1$. However, unlike the industry practice of volatility freezing, the model can remain market consistent, although radically damped if the term rates’ total implied variances exceed predefined thresholds.

In order to mitigate the blow-ups of term rates, this section has put the focus on the damping factors so far. However, [2] observed in their simulation study that the probability of simulating unrealistically high term rates under the spot measure $\mathbb {Q}^*$ can also be greatly reduced by changing the term rates’ instantaneous correlation structure. Here, the maps $\left\{ v_i\right\} _{i=1}^N$ that model the term rates’ instantaneous correlations (see also Remark 2.2) provide the theoretical framework for this idea.

#### Example 2.3

(Decorrelation beyond a threshold) This choice of correlation factors is based on [2]. Let $1 \le i \le N$ and consider a model with $d=N$-dimensional Brownian motion in the background. Moreover, let $1 \le d_1 \le d$ and $\left\{ u_i\right\} _{i=1}^{d_1}$ be the unit vectors in $\mathbb {R}^{d_1}$. The role of the later will be discussed in the next subsection. We call the choice of correlation factors of the form

$$\begin{aligned} v_i(x):= v_i(t,x) = {\left\{ \begin{array}{ll} \iota _N\left( u_i\right) &  \text {if } x \le \tau _1, \\ e_i &  \text {if } x > \tau _1, \end{array}\right. } \quad \quad (1 \le i \le d) \end{aligned}$$

*decorrelation beyond a threshold* where $\iota _N: \mathbb {R}^{d_1} \rightarrow \mathbb {R}^d$ denotes the canonical embedding of $\mathbb {R}^{d_1}$ in $\mathbb {R}^d$ and $\{e_i\}_{i=1}^d$ is the canonical orthonormal basis of $\mathbb {R}^d$. Observe that the *i*-th term rate’s dynamics in this example is driven by standard *d*-dimensional Brownian motion, although only standard $d_1$-dimensional Brownian motion is required if its total implied variance *x* does not exceed the threshold $\tau _1$.

#### Damping thresholds and market consistency

In this subsection, we discuss an important aspect of the model parametrisation, i.e. the choice of damping thresholds to ensure “market consistency” when damping the term rates’ instantaneous volatilities. Hereby, a market model is generally referred to as *market consistent*, if the model (approximately) yields the same prices as the market for the interest rate derivatives to which it has been calibrated. Of course, this is not a rigorous mathematical definition. Mathematically, we understand this important model feature in the following way:

##### Definition 2.4

Let $\left\{ L_i\right\} _{i=1}^N$ be undamped term rates that satisfy

$$\begin{aligned} {\left\{ \begin{array}{ll} \textrm{d}L_i(t) = L_i(t) g_i(t) \left( u_i\right) ^T \textrm{d}W_t^i \quad 0 \le t \le T_i, \\ L_i(0) = F\left( 0,T_{i-1},T_i\right) > 0, \end{array}\right. } \end{aligned}$$

for all $1 \le i \le N$. Moreover, let $\left\{ F_i\right\} _{i=1}^N$ be the damped term rates that solve (2). Then, these two models are *consistent* for the tenors ${T_1< \dots < T_K}$ (${1 \le K \le N}$) if the model prices of all caplets and swaptions defined on this subtenorstructure coincide.

##### Remark 2.4

Clearly, the two models induce different cap and swaption prices for caps and swaptions with tenors $T_{K+1}< \cdots < T_N$.

As mentioned before, we choose the same total implied variance structure, *V*, for all term rates. If we assume that ${k_0 = \textrm{id}_{\mathbb {R}^+_0}}$ and that the correlation structure of the damped model is chosen as described at the end of Sect. 2.2, we immediately conclude that the two models above are consistent iff

13 $$\begin{aligned} \tau _1 \ge \tau _{\min } := \max _{1 \le i \le K} \int _0^{T_i} g_i(s)^2 \textrm{d}s \end{aligned}$$

and

14 $$\begin{aligned} v_i(t,\phi _i(t)) = u_i \end{aligned}$$

for any $t \in \left[ 0,\phi _i^{(-1)}(\tau _1)\right]$ and for all $1 \le i \le K$. In order to observe a damping effect, note that $\tau _1 > 0$ must satisfy

$$\begin{aligned} {\tau _1 < \tau _{\max }:= \int _0^{T_{N}} g(s)^2 \textrm{d}s}. \end{aligned}$$

Although the idea presented in this subsection sets lower and upper bounds for the first damping threshold, it does not provide any insight into what would characterise a reasonable choice of damping factors and damping thresholds. One approach to solve this problem is presented in the Appendix.

From a calibration perspective, this allows us to separate calibration and damping and calibrate the classical Forward market model to the given market data at first. If this model sufficiently captures the market dynamics for the tenors $T_1< \dots < T_K$ for which cap and swaption prices are observed, so does the damped model if it satisfies (13) and (14). We say that such a damped model is *market consistent*.

## Calibration results

In order to demonstrate the applicability and effectiveness of the damping approach proposed in Sect. 2, we calibrate a market model to 1-year EURIBOR and ESTR OIS market data from May 1, 2023. More specifically, we model the evolution of the 1-year OIS rates $\{F_i(\cdot )\}_{i=1}^{60}$ by the (Mean-Field) Forward market model in (1). Denoting $\{E_i(\cdot )\}_{i=1}^{60}$ the 1-year EURIBOR rates, we then assume that $E_i(\cdot ) = F_i(\cdot ) + E_i(0) - F_i(0)$ for all tenors $1 \le i \le 60$. For discounting purposes, as is market standard, the OIS curve is used as a proxy for the risk-free forward rates, while the EURIBOR is the actual reference interest rate for any interest rate derivative. We then extrapolate from the given market data to simulate term rates from the damped model with maturities of 60 years in the future. The market data used to calibrate the model can be found in Tables 1, 2 and 3.

**Table 1:** 1-year EURIBOR forward rates as of May 1, 2023. *Source*: Refinitiv Eikon.

*T*	0Y	1Y	2Y	3Y	4Y	5Y	6Y	7Y	8Y	9Y
$\%$	3.036	3.9397	3.6789	3.4501	3.3305	3.2848	3.2671	3.2634	3.2737	3.2906

	10Y	11Y	12Y	15Y	20Y	25Y	30Y	40Y	50Y	
$\%$	3.3119	3.3289	3.3459	3.3419	3.1675	2.9508	2.7549	2.4613	2.228

**Table 2:** 1-year forward ESTR OIS rates as of May 1, 2023. *Source*: Refinitiv Eikon.

*T*	0Y	1Y	2Y	3Y	4Y	5Y	6Y	7Y	8Y	9Y
$\%$	2.9775	3.5647	3.2442	3.0167	2.885	2.8203	2.7863	2.7692	2.7684	2.7782

	10Y	11Y	12Y	15Y	20Y	25Y	30Y	40Y	50Y	
$\%$	2.7961	2.8175	2.8425	2.8749	2.773	2.611	2.4647	2.2517	2.0854

**Table 3:** 1-year EURIBOR/ESTR OIS ATM swaption volatilities (Black–Scholes), $\left\{ \sigma ^{\text {BS}}_{i,j-i}\right\} _{i < j}$, as of May 1, 2023. *Source*: Refinitiv Eikon.

%	1Y	2Y	3Y	4Y	5Y	6Y	7Y	8Y	9Y	10Y
1Y	37.55	41.56	40.18	36.98	34.65	32.73	30.48	28.92	27.50	26.75
2Y	41.27	41.95	39.43	36.56	34.25	32.16	30.05	28.54	27.45	26.58
3Y	41.02	40.26	37.80	35.11	32.85	30.76	28.70	27.48	26.48	25.90
4Y	39.89	38.61	36.26	33.67	31.49	29.51	27.73	26.61	25.88	25.48
5Y	38.55	37.05	34.74	32.28	30.25	28.55	26.95	26.03	25.43	25.16
6Y	37.40	35.89	33.73	31.41	29.47	27.84	26.44	25.67	25.24	
7Y	36.20	34.79	32.80	30.73	28.75	27.30	26.06	25.46		
8Y	34.99	33.85	32.00	29.99	28.21	26.91	25.82			
9Y	33.85	32.92	31.22	29.37	27.79	26.67				
10Y	32.62	32.00	30.49	28.83	27.41					

The y-axis’ index represents the expiry tenor $T_i$, while the x-axis’ index denotes the period to maturity, $T_j - T_i$

In our model, the forward rates are driven by the instantaneous volatilities

$$\begin{aligned} {\sigma _i(t) = g(T_{i-1}- t) \, f\left( \phi _{i}(t)\right) \, v_i\left( \phi _{i}(t)\right) \quad \, 2 \le i \le 60, \ 0 \le t \le T_{i-1}.} \end{aligned}$$

Specifically, we choose ${g(t) = (x_1 + x_2 t + x_3 t^2) \exp \left( - x_4 t\right) + x_5,}$ which is a classical Rebonato-type principal factor (cf. [10, Sec. 21.3]). Moreover,

$$\begin{aligned} {\phi _i(t) = V\left( \int _0^t g_i(s)^2 \textrm{d}s\right) }, \end{aligned}$$

*V* is the total implied variance structure, *f* its associated damping factor and $\left\{ v_i\right\} _{i=2}^{60}$ is a set of correlation factors. The damping factors are chosen as in Examples 2.1 and 2.2 with ${\epsilon = 0.01}$ and ${\delta = 0}$. In particular, this means that there is only one damping threshold $\tau _1 = \tau$ in both cases. In this chapter, we refer to this $\tau$ as “the” damping threshold. To test the effectiveness of the decorrelation-beyond-a-threshold method, $\left\{ v_i\right\} _{i=2}^{60}$ are either chosen as described in Example 2.3 with threshold $\tau$ or in such a way that they preserve the correlation structure fitted to the market data.

### Calibration

Let $\{0,1,\cdots ,60\}$ be the underlying tenor structure of the market models and denote by $K=15$ the index of the largest tenor which is considered in the calibration procedure. The market model is calibrated to the market data in the usual three step process:

1. To begin with, we fit the available initial forward rates, $\left\{ F_i(0)\right\} _{i}$ and $\left\{ E_i(0)\right\} _{i}$, to Nelson–Siegel type families by means of least squares (c.f. [4, Ch. 3.3]).
2. Secondly, the principle factor *g* is fitted to the swaption volatilities with time to maturity of 1 year which are, in fact, the Black–Scholes volatilities of 1-year EURIBOR caplets. Assuming that the damping threshold $\tau$ is larger or equal than the minimum permissible threshold $\tau \ge 1.4322$ in accordance with the Market Consistency Criterion (13), we obtain
  $$\begin{aligned} \left| \sigma ^{\text {BS}}_{i,i+1}\right| ^2 = \frac{1}{T_i}\int _0^{T_i}g(s)^2\textrm{d}s \quad (1 \le i \le 59). \end{aligned}$$
  This already determines the lengths of the instantaneous volatilities.
3. In the final step, the instantaneous correlations are fitted to the swaption data given the caplet volatilities to obtain the directions of the instantaneous volatilities. To guarantee that the correlation matrix is positive definite with positive entries and to avoid numerical instabilities when fitting the correlations to the market data, the forward rates’ instantaneous correlations, $\left\{ \rho _{i,j}\right\} _{1 \le i,j \le N}$, are parametrised by
  $$\begin{aligned} \rho _{i,j}&= \exp \Bigg (-\frac{|j-i|}{N-1}\Bigg (-\log \left( \rho _\infty \right) \\&\quad + \eta _1 \frac{i^2 + j^2 + i\,j - 3\,N\,i - 3\,N\,j +3\,i + 3\,j +2\,N^2 -N - 4}{(N-2)(N-3)} \\&\quad + \eta _2 \frac{i^2 + j^2 + i\,j - N\,i - N\,j - 3\,i - 3\,j + 3\,N + 2}{(N-2)(N-3)}\Bigg )\Bigg ) \end{aligned}$$
  for every $1 \le i,j \le N$ with $N=60$,
  $$\begin{aligned} 0 \le \eta _2 \le 3\,\eta _1 \end{aligned}$$
  and
  $$\begin{aligned} {0 \le \eta _1 + \eta _2 \le -\log \left( \rho _\infty \right) } \end{aligned}$$
  as proposed in [11, Sec. 1]. For further information regarding suitable correlation structures, the interested reader is referred to [1, Sec. 6.9]. Ideally, we would like to fit the correlation structure to the swaption prices that are implied by the market model. Unfortunately, this would require the evaluation of swaption prices by means of Monte–Carlo simulation in every iteration of the minimisation procedure. Therefore, we apply the common approximation (cf. [4, Chpt. 11.5])
  15 $$\begin{aligned} \left| \sigma ^{\text {BS}}_{i,j}\right| ^2 \approx \sum _{p,q=i+1}^j \frac{v_p(0)v_q(0)E_p(0)E_q(0)\rho _{p,q}}{\left( R^{\text {Swap}}_{i,j}(0)\right) ^2}\frac{1}{T_{i}}\int _0^{T_{i}}\left| \sigma _p(s)\right| \left| \sigma _q(s)\right| \textrm{d}s, \end{aligned}$$
  to estimate the parameters $\{\eta _1, \eta _2, \rho _\infty \}$. Hereby, $R^{\text {Swap}}_{i,j}(0)$ denotes the vanilla swap rate with payment dates $T_{i+1}, \dots , T_j$ given by
  $$\begin{aligned} R^{\text {Swap}}_{i,j}(0) = \sum _{l=i+1}^j v_l(0) E_l(0), \quad v_l(0) = \frac{P(0,T_l)}{\sum _{k=i+1}^j P(0,T_k)} \end{aligned}$$
  where $\left( P(0,T_k)\right) _{k=0}^{60}$ are the zero-coupon bond prices induced by the initial forward OIS rates. Since the correlation matrix is positive definite, we find $\left\{ u_i\right\} _{i=1}^{60} \subset \mathbb {R}^{60}$ such that $u_i^T u_j = \rho _{i,j}$ for every $1 \le i,j, \le 60$. Thus, the Market Consistency Criterion (14) reads $v_i(x) = u_i$ for every $0 \le x \le 1.4322$ and for all ${1 \le i \le 60}$.

#### Calibration results

**Fig. 3:**
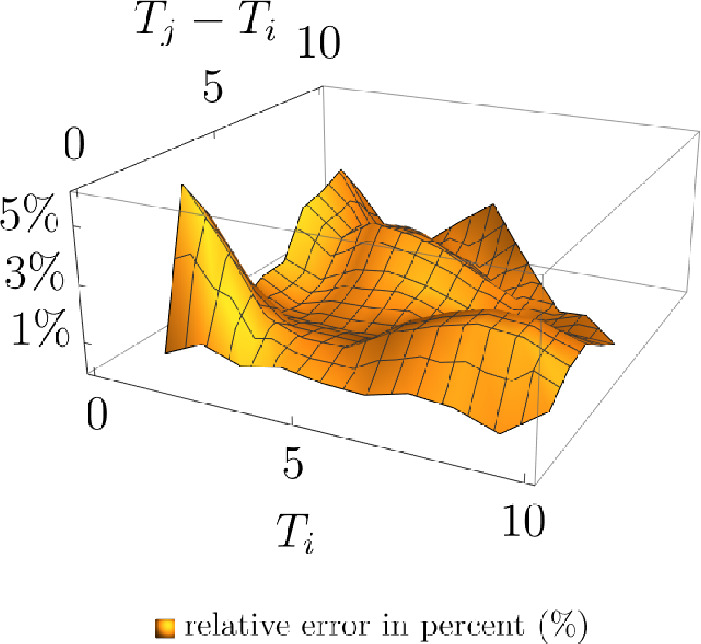
Model implied caplet volatilities with different damping approaches and various expiries

Fig. 3 suggests that the quoted implied caplet volatilities are well-matched by the (approximate) swaption volatilities of the market model. Indeed, $RMSE = 0.0051$. The fitted parameters can be found in Tables 4 and 5.

**Table 4:** Fitted principal factor parameters

$x_1$	$x_2$	$x_3$	$x_4$	$x_5$
$-0.0059$	0.2618	$-0.0481$	0.4002	0.2901

**Table 5:** Fitted correlation parameters

$\eta _1$	$\eta _2$	$\rho _\infty$
1.1040	0.1995	0.0020

### Dampening effects

To test the feasibility of the damping approach that is proposed in this paper, 3000 instances of $E_{60}(59) = F_{60}(59) + E_{60}(0) - F_{60}(0)$, the EURIBOR rate that is accrued in [59, 60], are simulated under the spot measure $\mathbb {Q}^*$. To this end, we apply the Euler-Mayurama scheme (cf. [4, Sec. 11.6]) to simulate the logarithmic OIS term rates: For every $1 \le i \le 60$, given instantaneous volatility processes $\left\{ \sigma _i\right\} _{i=1}^{60}$, Itô’s formula (cf. [4, Sec. 4.1]) implies

$$\begin{aligned} \textrm{d}\log \left( F_i(t)\right) = \sigma _i(t)^T \textrm{d}W_t^* + \sum _{j=\eta (t)+1}^i \frac{\Delta _j F_j(t)}{1+ \Delta _j F_j(t)} \sigma _i(t)^T \sigma _j(t) \textrm{d}t - \frac{1}{2}\left| \sigma _i(t)\right| ^2 \textrm{d}t \end{aligned}$$

with $\eta (t) = l$ iff $t \in (T_{l-1},T_l]$ for $1 \le l \le N$ under $\mathbb {Q}^*$. Thus, given an equidistant grid $0 = t_0< t_1< \cdots < t_N$ with ${t_{k+1} - t_k = h > 0} \ \ {\forall \, 1 \le k \le N}$, we can simulate $F_i$ at $t_k$ under the spot measure by

$$\begin{aligned} \log \left( F_i(t_k)\right)&= \log \left( F_i(t_{k-1})\right) + \sqrt{h}\,\sigma _i(t_{k-1})^TZ_{k-1} \\&\quad + \sum _{j=\eta (t_{k-1})+1}^i \frac{\Delta _j F_j(t_{k-1})}{1+ \Delta _j F_j(t_{k-1})} \sigma _i(t_{k-1})^T \sigma _j(t_{k-1}) h \\&\quad - \frac{1}{2}\left| \sigma _i(t_{k-1})\right| ^2 h \end{aligned}$$

${\forall \, 1 \le k \le N}$ and ${\forall \, 1 \le i \le 60}$. Here, $\left( Z_k\right) _{k=0}^{N-1}$ denotes a sequence of iid, 60-dimensional, uncorrelated Gaussian vectors with standard normally distributed marginals. For the purpose of this case study, $h = \frac{1}{10}$.

#### Simulation results

Before running large-scale simulations, we apply the ideas presented in the Appendix to predict the impact of our choice of damping thresholds: We are going to simulate $n=3000$ iid copies of the term rates $F_{60}(59)$ and we would like to choose the damping threshold $\tau$ such that the maximum of the simulated rates exceeds some $r > 0$ only with probability less than $p > 0$. For the purpose of this test, set $p = 20\%$. From Sect. 2.2.1 we recall the following:

$$\begin{aligned} \tau \ge \tau _\text {min} \quad&\implies \quad \text {market consistency}, \\ \tau < \tau _\text {max} \quad&\implies \quad \text {damping effect}. \end{aligned}$$

**Fig. 4:**
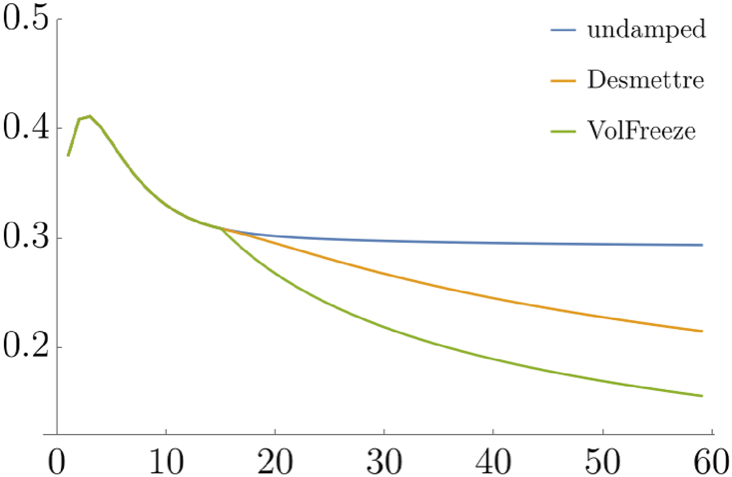
Approx. model swaption vol. versus quoted swaption vol. (ATM) with various expiries (x-axis) and periods to maturity (y-axis)

**Fig. 5:**
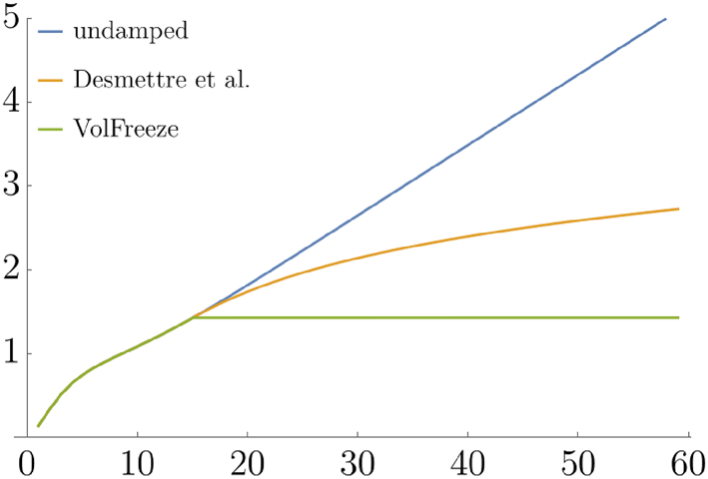
Model total implied caplet volatilities with different damping approaches and various expiries

In this example, $\tau _\text {min} = 1.4322$ and $\tau _\text {max} = 5.0976$. If we opt for the minimum permissible threshold $\tau _\text {min} = 1.4322$, Example A.1 shows that, for any $t \in [0,59]$, none of the 3000 simulated values of $F_{60}(t)$ under $\mathbb {Q}^{60}$ will exceed $r_\text {min} = 1.3002$, and $r_\text {min} = 3.8134$ will not be exceeded with probability $80\%$ if we apply the volatility freeze and the damping approach proposed by Desmettre et al. respectively. Furthermore, choosing $\tau _\text {max} = 5.0976$ increases this limit on the maximum of the 3000 simulated values of $F_{60}(t)$ under $\mathbb {Q}^{60}$ to $r_\text {max} = 11.5786$. Thus, we choose the minimum permissible threshold $\tau _\text {min}$ for the simulations under $\mathbb {Q}^*$. As mentioned in Sect. 2.2, these damping methods generally reduce the model’s implied caplet volatilities. A comparison of these extrapolated implied caplet volatilties can be found in Fig. 4 and Fig. 5.

**Fig. 6:**
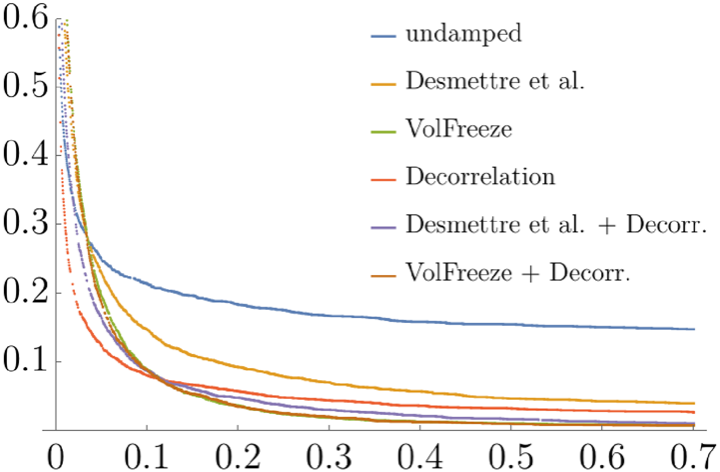
Empirical distribution’s tails of $E_{60}(59)$ with different damping approaches

Table 6 presents an overview of the relative number of extreme simulated values of $E_{60}(59)$ and Fig. 6 exhibits a comparison of the interest rate’s empirical distribution’s tails.

**Table 6:** Relative number of simulated values of $E_{60}(59)$ with respect to $\mathbb {Q}^*$ that exceed selected thresholds

*R*(59, 60)	$\ge 0.2$	$\ge 0.7$	$\ge 10^3$
Undamped	0.248	0.215	0.124
Decorrelation	0.057	0.026	0.0
Desmettre et al.	0.093	0.040	0.001
Desmettre et al. + Decorrelation	0.048	0.011	0.0
VolFreeze	0.036	0.008	0.0
VolFreeze + Decorrelation	0.035	0.007	0.0

Hereby, we find that reducing the instantaneous correlations alone can be a very effective method to reduce the number of blow-ups. However, when volatilities are extremely high, damping the instantaneous volatilities is still more effective. What is more, the volatility freeze and the combined method of volatility freeze and decorrelation leads to the least number of interest rate blow-ups, as expected.

## Summary

This contribution focuses on the mean-field extension of popular market models that are readily applied in the insurance industry. Since insurers are required to valuate their portfolios, some of which may be comprised of very long contracts, using arbitrage free interest rate scenarios, special emphasise is placed on the long-term extrapolation of term rate volatilities to obtain the best estimate of the market’s expectation regarding future interest rate movements.

In particular, we extend the work of [2] by developing a framework (see Sect. 2) that substantially reduces the computational effort required to simulate term rates from the given market models. Critically, this framework proves to be a minor complement to existing implementations of the Forward market model and can also be directly applied to in-arrears term rates stripped from short-term rates such as the ESTR. Furthermore, we strive to set out procedures and criteria to avoid arbitrariness with the theory developed in Sect. 2.2.1. Notably, this subsection presents criteria to avoid the radical damping or inflation of observed, valid caplet and swaption volatilities.

In Sect. 3, an exemplary simulation study was conducted based on the 1-year EURIBOR and ESTR OIS forward rates as of May 1, 2023 to test the feasibility of the proposed method. It is shown that the proposed damping approach considerably reduces the number of interest rate blow-ups while, crucially, preserving the term rates’ martingale property. Furthermore, these results are consistent with the results of the simulation study by [2], which identify damping of the instantaneous correlations as a very effective method for reducing the number of interest rate blow-ups. In addition, the (pseudo) volatility freeze proposed in Example 2.2 also proves to be a very powerful, albeit radical, damping approach that could be used in combination with other, less radical damping approaches as a last resort.

## Appendix A: Blow up probabilities

In this section, we propose a method that allows us to predict the effectiveness of a selection of damping thresholds in reducing the number of interest rate explosions before running large-scale simulations.

Suppose we would like to simulate *n* iid copies $\left\{ F_i^{(j)}(t)\right\} _{j=1}^n$ of the term rate $F_i(t)$ for large *i* and *t*. Given a suitable set of segments, it might be natural to choose the damping thresholds $\left\{ \tau _j^i\right\} _{j \in \mathbb {N}}$ in such a way that the maximum of the simulated term rates exceeds a threshold *r* only with predefined probability less than $p > 0$.

Using the notation of Corollary 2.1.1, recall that

$$\begin{aligned} \log \left( F_i(t)\right) \sim \mathcal {N}\left( \log \left( F_i(0)\right) - \frac{1}{2}\int _0^t \left| \lambda _i(s)\right| ^2 \, \textrm{d}s, \ \int _0^t \left| \lambda _i(s)\right| ^2 \, \textrm{d}s\right) \end{aligned}$$

under $$\mathbb {Q}^i$$ and

$$\begin{aligned} \phi _i(t) = \log \left( \frac{\mathbb {E}_{\mathbb {Q}^i}\left[ F_i(t)^2\right] }{F_i(0)^2}\right) = \int _0^t \left| \lambda _i(s)\right| ^2 \, \textrm{d}s. \end{aligned}$$

For this reason, we have to ensure that

$$\begin{aligned} 1 - p \le \mathbb {Q}^i\left[ \max _{1 \le j \le n} F_i^{(j)}(t) < r \right] = \Phi \left( \frac{\log \left( \frac{r}{F_i(0)}\right) + \frac{1}{2}\phi _i(t)}{\sqrt{\phi _i(t)}}\right) ^n \end{aligned}$$

where $\Phi$ is the CDF of the standard normal distribution. This inequality holds iff

A1 $$\begin{aligned} c_n(p) \sqrt{\phi _i(t)} \le \log \left( \frac{r}{F_i(0)}\right) + \frac{1}{2}\phi _i(t) \end{aligned}$$

with $c_n(p):= \Phi ^{(-1)}\left( \left( 1-p\right) ^\frac{1}{n}\right)$. Note that

A2 $$\begin{aligned} x^*_i := 2\left( c_n(p)^2 -\log \left( \frac{r}{F_i(0)}\right) - \sqrt{c_n(p)^4-2 \log \left( \frac{r}{F_i(0)}\right) c_n(p)^2}\right) \end{aligned}$$

is the lesser of the two roots satisfying

$$\begin{aligned} c_n(p)^2 x = \left( \log \left( \frac{r}{F_i(0)}\right) + \frac{x}{2}\right) ^2. \end{aligned}$$

If we choose sufficiently small $$0< p < 1$$ and sufficiently small $$r > F_i(0)$$, $${c_n(p)^2 > 2 \log \left( \frac{r}{F_i(0)}\right) }$$. In this case, $$x_i^* > 0 $$ and (A1) holds if

A3 $$\begin{aligned} \phi _i(t) \le x^*_i \quad \Leftrightarrow \quad \int _{T_{i} - t}^{T_{i}} g(s)^2 \textrm{d}s \le V^{(-1)}( x^*_i ). \end{aligned}$$

Clearly, not every choice of ${0< p < 1}$, $r > 0$, damping thresholds and damping segments that satisfy the conditions of Sect. 2.2.1 can fulfil this inequality. For this reason, it is advisable to first define the damping segments and the desired $0< p < 1$ and only then to choose $r > 0$ and the damping thresholds according to Sect. 2.2.1 and Equality (A3). Such an analysis is performed below for the damping factors introduced in Examples 2.1 and 2.2.

### Example A.1

(cont. of Examples 2.1 and 2.2)  

For arbitrary $\tau > 0$, fix $n>> 1$ and let

$$\begin{aligned} \left| \lambda _i(t)\right| = g\left( T_{i}-t\right) f\left( \phi _i(t)\right) \quad 1 \le i \le N \end{aligned}$$

where *f* is defined as in Example 2.1 or Example 2.2 with $\delta = 0$. In particular, this means that there is only one damping threshold $\tau _1 = \tau$ in both cases. Moreover, let *K* be the maximum tenor up to which market data is available. Here, we would even like to choose $\tau > 0$ such that ${1 - p \le \mathbb {Q}^i\left[ \max _{1 \le j \le n} F_i^{(j)}(t) < r \right] }$ for all $t \in [0,T_i]$ and $K < i \le N$. To ensure market consistency, we require

$$\begin{aligned} \tau \ge \tau _\text {min} = \max _{1 \le i \le K} \int _0^{T_{i}} g_i(s)^2 \textrm{d}s = \int _0^{T_{K}} g(s)^2 \textrm{d}s \end{aligned}$$

to fulfil the sufficient condition of Sect. 2.2.1. In order to observe a damping effect, recall that $\tau > 0$ must satisfy

$$\begin{aligned} {\tau < \tau _\text {max} = \int _0^{T_{N}} g(s)^2 \textrm{d}s}. \end{aligned}$$

Let $K < i \le N$ and recall $x_i^*$ from Eq. (A2).

In Example 2.1, Condition (A3) reads

$$\begin{aligned} \int _0^{T_{i}} g(s)^2 \textrm{d}s \le V^{(-1)}(x_i^*) = {\left\{ \begin{array}{ll} x_i^* &  \text {if } x_i^* \le \tau , \\ \frac{\tau }{2} + \frac{\tau }{2} \, \exp \left( \frac{2 (x_i^*- \tau )}{\tau }\right) &  \text {if } x_i^* > \tau . \end{array}\right. } \end{aligned}$$

Generally, we therefore require $\tau < x_i^*$ which reduces to

A4 $$\begin{aligned} \int _0^{T_N} g(s)^2 \textrm{d}s = \frac{\tau }{2} + \frac{\tau }{2} \, \exp \left( \frac{2 (x_i^*- \tau )}{\tau }\right) \end{aligned}$$

if we would like to choose the same damping threshold $\tau$ for all $K < i \le N$. In Example 2.2, Condition (A3) likewise reads

$$\begin{aligned} \int _0^{T_i} g(s)^2 \textrm{d}s \le V^{(-1)}(x_i^*) = {\left\{ \begin{array}{ll} x_i^* &  \text {if } x_i^* \le \tau , \\ \frac{x_i^* - (1 - \epsilon ^2) \tau }{\epsilon ^2} &  \text {if } \tau < x_i^*. \end{array}\right. } \end{aligned}$$

So, we require $\tau < x_i^*$ which again reduces to

A5 $$\begin{aligned} \int _0^{T_N} g(s)^2 \textrm{d}s = \frac{x_i^* - (1 - \epsilon ^2) \tau }{\epsilon ^2}. \end{aligned}$$

Given these constraints and some ${0< p<< 1}$ the question arises as to which $\tau$ and *r* one might choose. To this end, note that the right hand sides of (A4) and (A5) decrease monotonically in $\tau$ for fixed *r* and monotonically increase in *r* for fixed $\tau$. These observations agree with our intuition, because the smaller $\tau$, the larger the damping effect and the smaller the (minimal) threshold rate *r*. If we denote the minimally permissible and maximally meaningful threshold rates *r* by $r_{i, \text {min}}$ and $r_{i, \text {max}}$ respectively and interpret $x_i^*$ as a function of *r*, we obtain

$$\begin{aligned} x_i^*\left( r_{i,\text {min}}\right)&= \int _0^{T_{K}} g(s)^2 \textrm{d}s + \frac{1}{2}\left( \int _0^{T_{K}} g(s)^2 \textrm{d}s\right) \log \left( 2\left( \frac{ \int _0^{T_i} g(s)^2 \textrm{d}s}{\int _0^{T_{K}} g(s)^2 \textrm{d}s}\right) -1\right) , \\ x_i^*\left( r_{i,\text {max}}\right)&= \int _0^{T_N} g(s)^2 \textrm{d}s \end{aligned}$$

in Example 2.1 and

$$\begin{aligned} x_i^*\left( r_{i,\text {min}}\right)&= \epsilon ^2 \int _0^{T_i} g(s)^2 \textrm{d}s + (1 - \epsilon ^2) \int _0^{T_{K}} g(s)^2 \textrm{d}s, \\ x_i^*\left( r_{i,\text {max}}\right)&= \int _0^{T_N} g(s)^2 \textrm{d}s \end{aligned}$$

in Example 2.2. Denoting $x^*_{i,\min }:= x_i^*\left( r_{i,\text {min}}\right)$ and ${x^*_{i,\max }:= x_i^*\left( r_{i,\text {max}}\right) }$ and solving these equations for $r_{i,\text {min}}$ and $r_{i,\text {max}}$ yields

$$\begin{aligned} r_{i,\text {min}}= &   \exp \left( c_n(p) \sqrt{x^*_{i,\min }} - \frac{x^*_{i,\min }}{2} \right) F_i(0),\\ r_{i,\text {max}}= &   \exp \left( c_n(p) \sqrt{x^*_{i,\max }} - \frac{x^*_{i,\max }}{2} \right) F_i(0) \end{aligned}$$

assuming $c_n(p)^2 \ge x_{i, \max }^*$. Thus, the globally minimally permissible and maximally meaningful threshold rates are $r_{\text {min}} = \underset{K < i \le N}{\max }\ r_{i,\text {max}}$ respectively $r_{\text {max}} = \underset{K < i \le N}{\min }\ r_{i,\text {max}}$.

The inequality $c_n(p)^2 \ge \underset{K < i \le N}{\max }\ x_{i, \max }^*$ defines an upper bound for *p* which is usually almost 1, even for moderately small $n \in \mathbb {N}$. Furthermore, given such *p*, for every ${r \in \left[ r_{\text {min}},r_{\text {max}}\right) }$ $x^*(r)$ is well-defined and there exists an unique $\tau (r) > 0$ such that Eqs. (A4) and (A5) are satisfied for all $K < i \le N$. In the examples given, $\tau (r)$ can easily be calculated analytically.

## Footnotes

1 For further information regarding the Solvency II framework see https://www.eiopa.europa.eu/browse/regulation-and-policy/solvency-ii_en (Accessed: May 12, 2023).

2 I.e. $\mathcal {F}$ is right-continuous and $\mathbb {Q}^N$-complete.

3 $\text {id}_{\mathbb {R}_0^+}$ refers to the identity map on $\mathbb {R}_0^+$.
